# PROTOCOL: Arts‐based interventions for offenders in secure criminal justice settings to improve rehabilitation outcomes: An evidence and gap map

**DOI:** 10.1002/cl2.1255

**Published:** 2022-08-23

**Authors:** Jacqueline Tallent, Jes Phillips, Esther Coren

**Affiliations:** ^1^ Sidney De Haan Research Centre for Arts and Health Canterbury Christ Church University Canterbury Kent UK

## Abstract

This is the protocol for a Campbell review. The objective of this evidence and gap map is to presents the existing research on the impact of arts‐based interventions in secure criminal justice settings (SCJS) that aim to improve desistance outcomes for offenders. It will indicate the quality of available evidence, highlighting the gaps and informing future research priorities. Importantly, it will also identify where the evidence could be systematically reviewed. This would clearly produce a more comprehensive understanding of the available knowledge and an opportunity to move forward in a more direct and focussed way, with the potential to influence research, intervention development, and inform funding decisions.

## 1 BACKGROUND

### 1.1 Introduction

#### 1.1.1 The problem, condition or issue

According to Global Prison Trends, 2020, the global prison population is estimated to be more than 11 million, a record level to date, and recidivism rates, although not collected universally, are alarmingly high in some regions. For example, in England and Wales, it is estimated that half of all crime is committed by those who have previously offended (Ministry of Justice, 2015). In Ireland in 2010, 45% of people in the criminal justice system (CJS) reoffended within 3 years (Central Statistics Office, 2016). In Sweden, 39% of prisoners who offended in 2013 reoffended within 3 years. For those with nine or more previous adjudications, the likelihood of reoffending goes up to 92% (Brå, 2017). In America, 76.6% of offenders are re‐arrested within 5 years (Durose et al., 2014).

These high reoffending rates can have lasting social consequences (United Nations, 2012). Societal inequalities, such as adverse childhood events (ACEs), unemployment, poor educational achievement, poverty, social exclusion, mental and physical health, being a child in out‐of‐home care, and family breakdown, are common factors that can influence an individual's engagement in criminal behaviour (Beresford et al., 2020; Duque & Mcknight, 2019; Eaglesham et al., 2017; Webster & Kingston, 2014). Houchin, 2005 researched deprivation and imprisonment rates in Scotland using address data from those in prison at that time. Their results showed that the imprisoned population disproportionately comes from the most deprived communities in Scotland, particularly demonstrating that the probability of imprisonment increases with increasing deprivation. This is not to say all those living in poverty will commit a crime, however, when there is a combination of complex factors being experienced at once, the likelihood of criminal behaviour increases (Houchin, 2005; Webster & Kingston, 2014). Shepherd and Purcell (2015) researched the factors associated with criminal behaviour among young people with mental health problems. Their results demonstrate that the common risk factors among the general population, such as poor attendance at school, unemployment, substance use and adverse life events, are also associated with offending among young people with mental health problems.

Once people are in prison, they experience disproportionate health inequalities compared to the general population. The World Health Organisation (2021) recognises the harmful impact of imprisonment on mental health through lack of privacy, overcrowding, isolation from family and friends, enforced solitude, lack of purpose and inadequate health services, particularly mental health services. Higher levels of mental illness, self‐harm and suicide are experienced by those within the CJS (Fazel et al., 2017; Ministry of Justice, 2015; National Statistics, 2021). Some studies show suicide rates in prisons to be three times higher for men and nine times higher for women (Fazel et al., 2011, 2017), and in England and Wales, self‐harm reached its highest point in the 12 months leading up to March 2017 (Ministry of Justice, 2015). Longitudinal research conducted in Sweden suggests the presence of a psychiatric disorder can be associated with a substantially increased risk of violent reoffending (Chang et al., 2015). The Covid‐19 pandemic has rapidly impacted the deterioration prisoners' mental healthdue to greater prolonged periods of isolation and solitary confinement, reduced contact with staff, and suspended prison visits. These factors have been linked indirectly to reduced recidivism (De Claire & Dixon, 2017; Hewson et al., 2020). The physical health of prisoners is also much poorer than that of the general population, with lower life expectancy and higher rates of communicable and chronic diseases such as tuberculosis, HIV, hepatitis C and diabetes (House of Common, 2018; Lawrence et al., 2007).

For people leaving prison and re‐entering their communities, the social, economic and health inequalities mentioned above persist. The Social Exclusion Unit (2002) identified nine key factors which influence the likelihood of reoffending: education, employment, housing, financial support and debt, substance use, mental and physical health, attitudes and self‐control, institutionalisation and life‐skills and family networks. A prison sentence for someone already experiencing social exclusion and inequalities may worsen their situation upon release, increasing the likelihood of reoffending. It is not uncommon for people to lose their employment, housing and contact with family during their time incarcerated (Social Exclusion Unit, 2002). This negative cycle, characterised by exclusion and inequalities perpetuated by incarceration, keeps vulnerable people in a situation that jeopardises their ability to live a healthy and fulfilling life and positively contribute to society.

A key priority for interventions with offenders is a reduction in reoffending behaviour. ‘Desistance’ from crime has become a meaningful way to think about reducing reoffending, to adopt a more humanising approach that promotes the value of the individual, who they are and their potential (McNeill et al., 2012). Maruna and Farrall (Maruna & Farrall, 2004, p.7) propose that ‘to desist from crime, ex‐offenders need to develop a coherent, pro‐social identity for themselves’. Such change refers to the individual making changes in which they embrace a positive identity, one where they can see themselves outside the restrictive label of 'criminal' (Caulfield et al., 2022; Davey et al., 2015; McNeill et al., 2011).

Some of the efforts addressing desistance have focused on the arts and how they can help individuals make more meaningful changes in their lives, leading to desistance from crime. Arts is being recognised for its therapeutic value, with interventions being found to improve emotional and mental health, self‐awareness, social skills, communication and emotional maturity (Coholic et al., 2020; Hu et al., 2020; Khadar et al., 2013). Arts‐based interventions are being used to address specific health conditions such as dementia, COPD, Parkinson's disease, and mental health problems (Clift et al., 2013; Coholic et al., 2020; Cucca et al., 2021; Liu et al., 2019; Shoesmith et al., 2021), and in different settings for example within CJS, hospitals, and schools (Caulfield et al., 2022; Kimport & Hartzell, 2015; McDonald & Holttum, 2020; McNeill et al., 2011). Evidence suggests that art‐based interventions within the CJS may improve wellbeing, behaviour, self‐esteem and incite introspection, which may contribute to desistance (Anderson et al., 2011; Brewster, 2014; Cheliotis & Jordanoska, 2016; Crossick & Kaszynska, 2016; Davey et al., 2015; Maruna, 2001; Nickeas, 2018). Arts‐based interventions have been seen as valuable for engaging offenders in purposeful activity, which addresses the offender's humanity and rehabilitation needs leading to a crime‐free life (Bilby et al., 2013; Cohen, 2012).

Art‐based interventions in secure criminal justice settings (SCJS) which include any secure criminal justice facility, all ranges of security level in any contained establishments, including psychiatric hospitals, prisons, jails, youth offender settings, and secure childrens' or old age care homes, continue to grow, as does the number of individual impact evaluations (Cohen, 2009; George & Kasinathan, 2015; Jabbari & Dadvar, 2018). There is, however, still ambivalence around the methods and quality of evidence produced and the influence it has on policy (Arts Council England, 2018; McAvinchey, 2017). It is, therefore, crucial that available evidence is summarised in a way that allows practitioners, researchers, organisations, funders, and policymakers to see what evidence currently exists, the quality and where the gaps are for a more strategic, joined‐up approach to future research priorities. As funding and resources are limited, this is particularly useful to enable research commissioners to direct funds to areas that need more attention. In the UK, the National Criminal Justice Arts Alliance, Arts Council England, and other organisations recognise the complex and fragile nature of delivering arts interventions to offenders and highlight the need for effective collaborations, a robust‐evidence‐base, and the role of research in developing practice and policy (Arts Council England, 2018; Plant & Dixon, 2019).

#### 1.1.2 The intervention

The intervention may be any art‐based intervention which may include music, creative writing, theatre performance, visual arts, movement, and multi‐arts. These activities may be delivered in individual or group settings, in person or online.

Arts‐based practices aim to bring about a positive affect experience on the participants, indirectly rather than directly impacting offending behaviour by gaining a sense of community, time passing at a different pace, and improving self‐satisfaction and achievement (Parkes & Bilby, 2010). There is also evidence to show that arts and culture in CJS can provide learning opportunities, enhance safety and wellbeing in prisons, improve prisoner relationships with each other and staff, and positively influence family connections and links with the outside (National Criminal Justice Arts Alliance, 2021), all of which may help create a sense of agency and contribute to a more positive sense of self.

#### 1.1.3 Why it is important to develop the evidence and gap map (EGM)

With the global prison population increasing, over 100 countries have reported above maximum capacity levels in their prisons (Global Prison Trends, 2020). Severe health inequalities have worsened due to the Covid‐19 pandemic, and reoffending rates have increased. It is crucial to have an organised, systematic, and purposeful research agenda which utilises resources effectively and efficiently and can be used on a global scale (Coates, 2016; McLewin, 2011). An EGM in arts and offender rehabilitation will offer a global knowledge base that presents existing research, highlights the gaps, and offers examples of research varying in quality, thus allowing future projects to address under‐researched areas directly. It will also help identify areas where there is potential for systematic reviews, help funding bodies direct resources accordingly and helps practitioners, organisations and researchers identify effective characteristics of past interventions to enhance their intervention or evaluation methods.

Art is increasingly recognised as of therapeutic value. Interventions have reported positive effects on emotional and mental health, self‐awareness, social skills, communication and emotional maturity (Coholic et al., 2020; Hu et al., 2020; Khadar et al., 2013). The importance of art‐based interventions is being recognised across different sectors and reflects in the growing body of evidence. The arts are well established within the CJS, making this EGM a timely map to plot the existing evidence, particularly regarding the potential indirect impact on desistance related outcomes via impacts as described above.

### 1.2 Existing EGMs and/or relevant systematic reviews

No current EGMs focus on improving desistance outcomes using arts‐based interventions among SCJS. The Arts Council England have prepared a summary of evidence for the arts and culture within the CJS (Arts Council England, 2018). This document focuses on the value of arts research in achieving desistance and identifies several useful resources which will help inform the present EGM.

A small number of systematic reviews have been conducted in this field, however, they are either outdated, have a different scope, or different inclusion criteria. Meekums and Daniel (2011) conducted a systematic review to understand if the arts can have a therapeutic value for offenders, which, although relevant, is now 10 years old. Coutinho et al. (2015a, 2015b) carried out a systematic review to evaluate the evidence on active music‐making interventions for adults in SCJS, including correctional and forensic psychiatry facilities at various security levels, however, the review is exclusively concerned with music and no other art forms. Cheliotis and Jordanoska (2016) conducted a critical review of the evidence to understand to what degree facilitated art programmes contribute to desistance from crime. This has a similar scope but is not a systematic review, so it does not meet the set standards. Most of the current studies within this field call for more research. An EGM will concisely describe the existing research and highlight which areas should be the focus of future research.

## 2 OBJECTIVES

This EGM presents the existing research on the impact of arts‐based interventions in SCJS that aims to improve desistance outcomes for offenders. It will indicate the quality of available evidence, highlighting the gaps and informing future research priorities. Importantly, it will also identify where the evidence could be systematically reviewed. This would clearly produce a more comprehensive understanding of the available knowledge and an opportunity to move forward in a more direct and focussed way, with the potential to influence research, intervention development, and inform funding decisions. Other objectives of the EGM include:

- Identifying gaps in the evidence which can inform future research.
  ∘ Understanding the availability of knowledge about what specific types of arts‐based interventions have success in engaging offenders.
  ∘ Understanding what is known about which arts‐based interventions may address areas of desistance.
- Identifying where primary research has been conducted and where future synthesis could be developed.
  ∘ Summarising the types of study design commonly used and what types of methods are utilised for collecting and analysing data and outcomes.
- Identifying, appraising, and summarising the existing evidence to influence research development.
  ∘ Considering whether the overall quality of evidence is sufficient to draw conclusions or if more needs to be done to improve the quality of evaluations.
- Contribute to the theory of change.
- Capture service user‐focused information on the desirability, acceptability, and accessibility of interventions and any information on process and implementation factors.

## 3 METHODS

### 3.1 The EGM: Definition and purpose

The International Initiative for Impact Evaluation [3ie] (3ie, 2021) states that EGMs are designed to be a valuable tool for ‘development decision‐makers, researchers and donors looking for evidence to inform programme investments and identifying where there is an urgent need for more research or rigorous evaluation’. Unlike most EGMs which focus solely on quantitative data, this EGM also aims to include qualitative studies, as discussed below in the section on ‘types of study design’. Therefore, for this map to include all possible research, much of which is conducted using qualitative methods, and reach its full potential, an alternative to traditional methods for creating EGMs needs to be considered.

To map research on the ‘what’ and the ‘where’, this EGM will review systematic reviews and impact evaluations or studies of effectiveness. The studies to be included will measure intervention effectiveness by including designs with controls to establish the casual attribution; RCT, quasi‐experimental, regression discontinuity, difference in difference, cross‐sectional and others of similar design where an analysis is possible. The quality of the evidence will be reported within the EGM.

### 3.2 Framework development and scope

As in other policy areas, the availability of research evaluations measuring impact may be seen as a determining factor in building the case for arts‐based interventions in CJ settings, particularly during a time of economic scarcity and changing political climates. The current evidence base varies in quality and lacks cohesion. The difficulty for art practitioners and organisations evaluating art‐based interventions has been noted, including time, capacity, motivation, and standard of data collection (Maguire et al., 2019). The conceptual underpinning of the framework was developed through a review of the current literature and already developed theory of change models designed from current research projects (Clean Break, 2017; The Forgiveness Project, 2010).

Our intervention and outcome framework was developed and adapted based on the Intermediate Outcome Measurement Instrument (IOMI) toolkit developed by Burrowes et al. (2013) and Liddle et al. (2019). Using their seven psychological domains, we developed our framework, consulted with the stakeholder group, reviewed published systematic reviews, and examined key policy documents (All‐Party Parliamentary Group on Arts, 2017; House of Common, 2018; Ministry of Justice, 2015). Liddle et al. (2019) developed and tested ‘a robust but user‐friendly instrument for the measurement of intermediate outcomes—these are outcomes that are directly or indirectly associated with reductions in reoffending over the longer term, and that in the short term indicate positive changes along an offender's pathway towards an offence‐free future’. This measurement is part of a toolkit, including a questionnaire and guidance on its use, which was developed through seven stages of research, including a review of the literature, consultation with stakeholders and detailed analysis of pilot data. The questionnaire developed has 29‐items based on seven psychological dimensions: resilience, agency/self‐efficacy, hope, wellbeing, motivation to change, impulsivity/problem‐solving and interpersonal trust. The toolkit offers a description of each dimension (this can be found in the conceptual framework section below), an example of what no or negative changes may look like and examples of what positive changes may look like. It should be noted that Liddle et al.'s original framework incorporated eight dimensions, however, the eighth dimension ‘practical problems’ was excluded as it covered areas relating to challenges faced after release (i.e., money, employment/prospects, health and fitness, housing, drugs, alcohol, relationships, and gambling.)

The framework will follow the traditional intervention‐outcome matrix where rows are intervention categories (or subcategories), and columns are the IOMI rehabilitation outcomes. Where reported we will also extract any information on service user perspectives and process and implementation factors.

### 3.3 Stakeholder engagement

The central team (JT and EC) will meet weekly to discuss the direction and scope of the EGM. The stakeholder advisory group consists of professionals and service users from all aspects of the CJS. Members include:

- Professor of Mental Health specialising in forensic psychiatry research.
- Professor of Forensic Psychology specialising in evaluating the impact of ‘alternative’ activities in prisons.
- Consultant forensic psychiatrist with clinical and research experience.
- Associate Professor of Sociology and Criminologist specialising in arts‐based research within prisons.
- Creative Programmes Director from a prison charity.
- Managing Director of a prison arts charity with experience of facilitating prison and community art programmes.
- Independent consultant who specialises in arts and criminal justice research and policy development.
- Various service users with lived experience of being in SCJS and engaging with art‐based interventions.

The members of this group were given the opportunity to comment on a summary of the proposed project which included a detailed explanation of the framework and theory of change model. Feedback from stakeholders has further defined and clarified aspects of the protocol as well as redefined or reordered outcomes of interest.

Stakeholders will continue to be engaged at different time‐points in the development of the EGM.

### 3.4 Conceptual framework

This EGM will document how arts‐based interventions in SCJS impact the intermediate outcomes for personal, interpersonal and community change that may contribute to life improvement and reduction in offending (Clean Break, 2017; Plant & Dixon, 2019; The Forgiveness Project, 2010). It is important to note that changes to offending behaviour is not a straightforward chain of events. The theory of change model suggests that arts‐based intervention may contribute to personal changes at an individual level, indirectly impacting other dimensions of their functioning.

**Figure 1:**
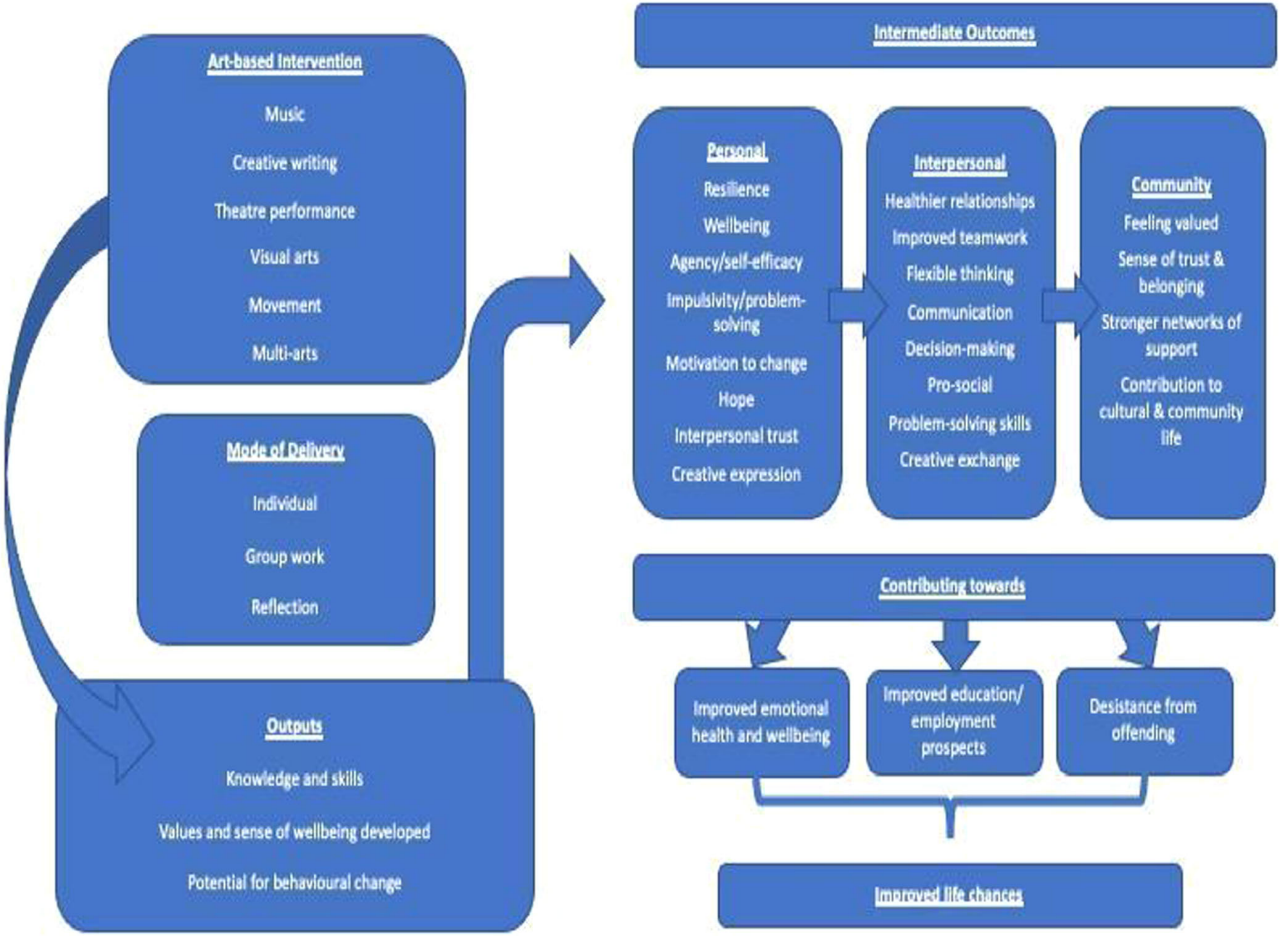
Theory of change

The theory of change model and conceptual framework for this EGM can be found in Figure 1. The arts‐based interventions act as the input which leads to intended intermediate outcomes.

### 3.5 Dimensions

The intervention‐outcome framework is based on the Intermediate Outcomes Measurement Instrument (IOMI) developed by Liddle et al. (2019) described above. The outcomes axis of the framework for this EGM will be based on the seven dimensions they found, which are designed to measure change; resilience, agency/self‐efficacy, hope, wellbeing, motivation to change, impulsivity/problem‐solving and interpersonal trust. The intervention categories are based on the six categories of arts‐based intervention: music, creative writing, theatre performance, visual arts, movement, and multi‐arts.

Within Liddle et al.'s toolkit, a description of each dimension is offered:

- Resilience—Capacity to recover from adversity, to move on positively or begin again. Related to individual coping skills and broader relationships and support networks.
- Agency/self‐efficacy—Whether one can make autonomous decisions about one's own life and make things happen in the outside world as a result.
- Hope—A calculation about perceived scope for positive future change, linked to motivation and self‐assessments of efficacy.
- Wellbeing—General or overall mental/emotional/psychological health or balance, linked to positive self‐regard and confidence.
- Motivation to change—A key focus is on internal rather than external motivation, linked to positive engagement.
- Impulsivity/problem‐solving—Lack of reflection and planning and a disregard of the consequences of behaviour. Highly impulsive people also generally lack problem‐solving skills.
- Interpersonal trust—Positive attitudes toward and connectedness with others. Links to notions of social capital.

Subcategories of each domain based on Liddle et al. (2019):

- Resilience—Coping skills, relationships/support networks.
- Wellbeing—Self‐perception, self‐worth, increased confidence levels.
- Agency/self‐efficacy—Independent decision‐making, locus of control, empowerment.
- Impulsivity/problem‐solving—Make conscious choices, increased planning, increased focus and discipline.
- Motivation to change—Positive engagement, attendance, internal motivation.
- Hope—Positive perception of future, higher levels of agency, higher levels of motivation.
- Interpersonal trust—Positive attitude towards others, connectedness/getting along with other prisoners, feeling part of a group, communication skills.

We will also code any information related to process and implementation factors noted in the studies.

#### 3.5.1 Types of study design

The EGM will include impact evaluations and systematic reviews of the effectiveness of interventions that aim to improve offenders' rehabilitation outcomes within SCJS. An impact evaluation will be defined as any intervention evaluation or field experiment that uses qualitative, quantitative, or mixed‐methods approaches applied to experimental or observational data that measures the effect of an intervention compared to what would happen to the same group in the absence of that intervention. Both completed and ongoing impact evaluations and systematic reviews will be included. To capture ongoing studies or reviews, trial registries and protocols will be searched. Authors will be contacted for a timescale of their project. If the timescale of data collection is outside the timescale for the project, it will not be included in the current EGM, but reference to the study will be noted for future updates.

Details of study design to be included:

1. randomised or non‐randomised design with comparison;
2. randomised control trials (RCTs) with assignment at the individual, setting, or institutional level; or
3. non‐RCTs using a quasi‐random method of prospective assignment (e.g., alternation of clusters); or
4. natural selection or allocation studies; or
5. controlled before‐and‐after studies (where the intervention and outcome measurements are contemporaneous)
6. ethnographic, phenomenological, observational, or narrative studies; or
7. studies explicitly described as systematic reviews and that describe methods used for search, data collection, and synthesis will be coded in the EGM and their included studies assessed for inclusion in the EGM.

The reasoning behind including non‐randomised designs was based on the knowledge that, typically, interventions in this field are delivered by service providers rather than researchers. As such, evaluation design may align with the service provision requirements rather than research standards.

This EGM will include impact evaluations where the comparison/control group receive no intervention (standard arts‐based intervention), a different intervention (e.g., psychological therapy), or a waiting list approach.

Any systematic reviews that summarise data on the effectiveness of arts‐based interventions to improve desistance outcomes for people in SCJS will be included in this EGM and their included studies will also be assessed for inclusion. Among the primary studies, RCTs provide the best evidence of effectiveness, however, the researchers are aware of the limited number of RCTs on arts‐based interventions within SCJS, and therefore will also be mapping non‐RCTs to enable the development of a complete map. Intervention evaluations using quantitative, qualitative, or mixed‐methods research within evaluation designs will be included if they match the primary outcome aims.

If there is ambiguity in the authors' description of the study design they have utilised, we will consult their methods section to assess the detail of what was done before assigning a design category. Where this remains unclear, we will contact the authors for further information.

For the inclusion of qualitative research, please see the section below.

#### 3.5.2 Treatment of qualitative research

This EGM will include qualitative research if it fits within the intervention/outcome framework and meets the inclusion and exclusion criteria. We will include projects that have been undertaken as part of a mixed‐methods study or studies that are entirely qualitative. Traditional methods such as ethnography, focus groups, or individual interviews will be considered for this EGM. Other forms of qualitative data collection methods such as documentation through observation, participatory designs, or written evaluation feedback will also be included in the EGM.

We will also include all research designs where data is collected on the views and experiences of service users or service providers, which relate to either barriers or facilitators to the effectiveness of arts‐based interventions and also the acceptability and accessibility of interventions. We will seek data that enables a more detailed understanding of why an intervention does (or does not) work as intended, for whom and in what circumstances. We will describe the characteristics of these studies in terms of the methods used to capture data on barriers and facilitators to and acceptability and accessibility of intervention implementation; the number of interviews/focus groups/observations that have taken place, who participated and the nature of qualitative data collection (type and time taken).

We will exclude discussion and opinion pieces, and single‐case papers.

#### 3.5.3 Types of intervention/problem

The present EGM will include interventions that aim to address people's rehabilitation outcomes within any SCJS using all/any arts‐based interventions. The studies to be included will be any systematic review or primary study that meets the inclusion criteria and key documents from a grey literature search.

The interventions may use any creative art form (e.g., music, creative writing, theatre performance, visual arts, movement, and multi‐arts) and will be aimed at all offenders of any age or gender and from any SCJS (i.e., prison, hospital). Interventions may use various delivery approaches, including individual or group work, facilitated by a professional or expert in a therapeutic environment. Intervention characteristics such as individual/group, delivery methods, and facilitators' details will be coded within our framework.

Comparisons will be included and classified as no intervention or treatment as usual (TAU). Where studies implement an arts‐based intervention combined with another form of intervention (e.g., lyric writing and psychoeducation), we will include data for the arts‐based intervention if presented separately within the study. If data are not presented separately, we will contact study authors to request disaggregated data. Where this is not available, the study will be excluded.

#### 3.5.4 Types of population (as applicable)

Study participants will include youth offenders (aged under 18), adult offenders (aged 18–65) and elderly offenders (aged 65+). The types of offenders included will be separated into preconviction offenders (or remand prisoners), sentenced prisoners, mental health offenders (either within a prison or in a secure hospital setting), and personality disordered offenders (either within a prison or secure hospital setting). The SCJS, as stated above, will include any secure criminal justice facility, all ranges of security level in any contained establishments, including psychiatric hospitals, prisons, jails, youth offender settings, and secure childrens' or old age care homes. The varying populations and facilities will be categorised separately and stratified by age, gender, ethnicity, disability, offender type and type of institution.

We plan to exclude any papers where the outcome is not directly focused on the service users.

If a study meets the inclusion criteria, but only a subset of the population is eligible for inclusion (e.g., some participants are in prison and some in the community), we will include only the eligible population if the data are disaggregated. Where the data are combined within the study, we will contact study authors to request the relevant disaggregated data. Only where we are unable to obtain such data in these ways will we exclude the study.

In the interests of capturing equity considerations, where the information is reported in the studies, we will include data on characteristics such as age, gender, ethnicity, sexual orientation, disability status, mental health status. Where such data are not reported, we will report the absence of the information in the gap map.

#### 3.5.5 Types of outcome measures (as applicable)

This study aims to scope the impact of arts‐based interventions. The primary outcome of interest for this EGM is the impact on the individual, based on outcomes relevant to desistance, measured at an individual level. Where studies measure an outcome but do not report it, we will seek the relevant outcome data from the study authors.

Relevant impact at an individual level will be analysed in our EGM using the Intermediate Outcomes Measurement Instrument (IOMI) developed by Liddle et al. (2019), as discussed above. We will include any outcome measure that captures any of these domains or other outcomes related to reoffending and/or desistance.

Any data reported on adverse or unintended effects of interventions will be extracted and coded into the map.

Data on process and implementation factors, as well as service user perspectives, will also be captured.

#### 3.5.6 Other eligibility criteria

None applicable

##### Types of location/situation (as applicable)

Not applicable

##### Types of settings (as applicable)

Only studies reporting interventions in SCJS will be included in the EGM. The types of study settings will be offenders detained within a SCJS (i.e., forensic psychiatric hospitals or correctional facilities, including youth offending centres, secure children's and old age homes).

### 3.6 Search methods and sources

This EGM will search for and include completed and ongoing primary studies and systematic reviews. The included studies of the systematic reviews will be assessed for eligibility of inclusion. This EGM will include both published and unpublished studies with no language or date restrictions to minimise publication bias. For studies written in a language other than English, we will attempt to obtain a complete translation.

The search strategy will aim to be an exhaustive search capturing published, unpublished, ongoing, and ‘grey’ literature (see Supporting Information: Appendix 2) To minimise discipline bias when searching databases, we will extend the search strategy to include other health, social care, and criminology journals and databases. We will seek advice from the various discipline‐specific librarians within the University library.

The following databases will be used to identify the completed and ongoing studies:

- Academic and trial registries:
  ∘ PsycINFO (via Ovid)
  ∘ EMBASE (via Ovid)
  ∘ MEDLINE (via Ovid)
  ∘ International Bibliography of Social Science (IBSS) (via ProQuest)
  ∘ Cumulative Index to Nursing and Allied Health Literature (CINAHL) (via EBSCO Host)
  ∘ National Institute for Health and Care Research (NIHR): https://www.journalslibrary.nihr.ac.uk/#/
  ∘ ClinicalTrials.gov
  ∘ International Clinical Trials Registry Platform (WHO): https://www.who.int/clinical-trials-registry-platform
  ∘ SCOPUS Preview (via Elsevier)
  ∘ Criminal Justice Abstracts (EBSCOhost)
  ∘ Dissertation and Theses Global (ProQuest)
  ∘ Social Science Citation Index (Web of Science)
  ∘ Arts & Humanities Citation Index (Web of Science)
  ∘ Conference Proceedings Index: Social Sciences & Humanities (Web of Science)
  ∘ CINCH: Australian Criminology Database (Informit)
- Systematic review databases:
  ∘ Epistemonikos: https://www.epistemonikos.org/
  ∘ Cochrane Library: https://www.cochranelibrary.com/
  ∘ Campbell Library: https://www.campbellcollaboration.org/better-evidence
  ∘ Internation Initiative Impact Evaluaiton(3ie): https://www.3ieimpact.org/
  ∘ EPPI centre: https://eppi.ioe.ac.uk/cms/
- We will search the repositories of organisations and conferences in the field of arts and health, criminal justice, or forensic organisation who are known to produce effectiveness evaluations on rehabilitation interventions to identify any ‘grey’ literature:
  ∘ American Correctional Association (ACA): https://www.aca.org/
  ∘ Home Office Research, Development and Statistical Archive: https://www.gov.uk/government/organisations/home-office/about/research
  ∘ Ministry of Justice (MoJ): https://www.gov.uk/government/organisations/ministry-of-justice
  ∘ National Institute of Corrections: https://nicic.gov/
  ∘ National Criminal Justice Research Service (NCJRS): https://www.ojp.gov/ncjrs
  ∘ National Institute of Justice (NIJ): https://nij.ojp.gov/
  ∘ Swedish National Council on Crime Prevention: https://www.government.se/government-agencies/the-swedish-national-council-for-crime-prevention/
  ∘ Department of Health and Social Care: https://www.gov.uk/government/organisations/department-of-health-and-social-care
  ∘ National Health Service (NHS): https://www.nhs.uk/
  ∘ Her Majesty's Prison & Probation Service (HMPPS): https://www.gov.uk/government/organisations/her-majestys-prison-and-probation-service
  ∘ National Criminal Justice Arts Alliance: https://artsincriminaljustice.org.uk/
  ∘ National Endowment for the Arts: https://www.arts.gov/
  ∘ Repository for Arts and Health Resources by Sidney De Haan Research Centre: https://research.canterbury.ac.uk/sidney-de-haan-research-centre-for-arts-and-health/repository-for-arts-and-health-resources/
  ∘ Prison Arts Resource Project: https://scancorrectionalarts.org/
  ∘ GoogleScholar: https://scholar.google.com/
  ∘ U.S. Office of Juvenile Justice and Delinquency Prevention: https://www.ojjdp.gov/mpg/litreviews/Arts-Based-Programs-for-Youth.pdf
  ∘ Prison Arts Resource Project: scancorrectionalarts.org
  ∘ crimesolutions.gov
  ∘ Crime Reduction Toolkit: http://whatworks.college.police.uk/toolkit/Pages/Toolkit.aspx
- Bibliographic searches:
  ∘ Bibliographic reference harvesting from the reference lists of all included primary studies and systematic reviews will be undertaken.
- Additional outreach:
  ∘ The authors of the included studies, key organisations, stakeholders, and personal contacts from the researchers' networks will be contacted to invite additional reports or ongoing studies.

### 3.7 Analysis and presentation

#### 3.7.1 Dependency

The unit of analysis for this EGM is the included studies (i.e., systematic reviews, primary studies of effectiveness and any ‘grey’ literature). Where multiple papers are published from the same study, the most recent open access publication will be included in the EGM. If previous publications of the same study include different outcome measures, these papers will be included only to report on the missing outcomes. If this arises, all publications from the same study will be treated as one single study. The final EGM will identify the number of studies covered by the map and list those studies with multiple papers clearly within the references.

#### 3.7.2 Report structure

The EGM report will synthesis the findings of the EGM, discuss gaps in the evidence, and highlight future work recommendations. A plain language statement of the EGM findings will also be created.

#### 3.7.3 Filters for presentation

The EGM will have two primary dimensions: interventions (rows) and outcomes (columns). A conceptual framework and PRISMA flow diagram will be included, and an online interactive matrix displaying the interactions between the intervention categories (or subcategories) and outcomes. We will use bubbles of varying sizes to present included studies. Different colours will be used for different types of studies (i.e., systematic reviews, primary studies of effectiveness and any ‘grey’ literature and qualitative studies). Searchable filters will include demographic information (gender, ethnicity, age), institutional settings (including security level), population information (type of offender, sentence classification), geography (country or region), study design (RCT, non‐RCT, mixed‐method, qualitative), study status (completed, ongoing).

### 3.8 Data collection and analysis

#### 3.8.1 Screening and study selection

The database searching will follow the traditional method of two independent researchers (J. T. and E. C.) to search and screen the title/abstracts to decide which items should be retrieved in full text. Two independent reviewers will also undertake full‐text screening of items that appear to meet inclusion criteria. Disagreements will be resolved by consensus. Automation or text‐mining will not be used in this EGM.

#### 3.8.2 Data extraction and management

A standardised data extraction and coding form will be used for primary studies to extract the descriptive data from all the studies included in the map (see Supporting Information: Appendix 3). The data to be extracted will include the following: bibliographic details, intervention types and descriptions, outcome types and descriptions, study design, and implementation details. Additional study characteristics, including country, data years, publication type, unit of analysis, study design, and study quality, will also be coded. This tool will be piloted to ensure consistency in coding and to resolve any issues or ambiguities. The data extraction process will run similarly to the screening process; two independent researchers (J. T. and E. C.) will conduct the data extraction for each study. Any disagreements will be resolved by consensus.

### 3.9 Tools for assessing risk of bias/study quality of included reviews

#### 3.9.1 Quality appraisal

All primary studies and systematic reviews will be assessed for risk of bias, quality or confidence using the most appropriate tool. Systematic reviews will be appraised using AMSTAR2 (Shea et al., 2017), primary research studies will be appraised using the Cochrane Risk of Bias 2 tool (Higgins et al., 2019) Non‐randomised studies will be coded using the ROBINS‐ I tool (Sterne et al., 2016). Qualitative, process and implementation studies will be assessed using a tool developed by Campbell (Keenan & White, 2018). These assessments will be completed independently by two study researchers (J. T. and E. C.), and any conflicts will be resolved by consensus.

#### 3.9.2 Methods for mapping

Two pieces of software developed by the EPPI Centre at the Social Science Research Unit of the UCL Institute of Education, University of London, UK, will be used in this review. We will use EPPI‐Reviewer 4 software (Thomas et al., 2010) to screen and code all eligible studies for inclusion into the map, and the interactive map will be developed using the EPPI‐Mapper (Digital Solution Foundry of EPPI Centre, 2020).

## CONTRIBUTIONS OF AUTHORS

- Content: JT, JP and EC
- EGM methods: JT and EC
- Information retrieval: JT and JP

## DECLARATIONS OF INTEREST

No conflict of interest.

## PLANS FOR UPDATING THE EGM

Once completed, the EGM will be updated every 5 years, depending on funding. The lead author and co‐authors will be responsible for updating the EGM.

## SOURCES OF SUPPORT

Internal sources

- Canterbury Christ Church University, Sidney De Haan Research Centre for Arts and Health, UK

Research Centre funding

External sources

- None, UK

## Supporting information

Supporting information.

## References

[cl21255-bib-0001] OTHER REFERENCES

[cl21255-bib-0002] International Initiative for Impact Evaluation (3ie) . (2021). *Evidence gap maps*. http://3ieimpact.org/en/evidence/gap-maps

[cl21255-bib-0003] All‐Party Parliamentary Group on Arts, Health and Wellbeing . (2017). *Creative Health: The Arts for Health and Wellbeing*. http://www.artshealthandwellbeing.org.uk/appg-inquiry/

[cl21255-bib-0004] Anderson, K. , Colvin, S. , McNeill, F. , Nellis, M. , Overy, K. , & Abd Tett, L. (2011). *Inspiring changes: Final project report of the evaluation team*. http://www.artsevidence.org.uk/media/uploads/evaluation-downloads.mc-inspiring-change-april-.pdf

[cl21255-bib-0005] Arts Council England . (2018). *Arts and culture in health and wellbeing and in the criminal justice system—A summary of evidence*. https://www.artscouncil.org.uk/publication/arts-and-culture-health-and-wellbeing-and-criminal-justice-system-summary-evidence

[cl21255-bib-0006] Beresford, S. , Loucks, N. , & Raikes, B. (2020). The health impact on children affected by parental imprisonment. BMJ Paediatrics Open, 4, 1–3. 10.1136/bmjpo-2018-000275 PMC7047477 32154384

[cl21255-bib-0007] Bilby, C. , Caulfield, L. , & Ridley, L. (2013). *Re‐imagining futures: Exploring arts interventions and the process of resistance*. http://www.artsevidence.org.uk/media/uploads/re-imagining-futures-research-report-final.pdf

[cl21255-bib-0008] Brewster, L. (2014). The impact of prison arts program in inmate attitudes and behaviour: A quantitative evaluation. Justice Policy Journal, 11(2), 1–28.

[cl21255-bib-0009] Brå, R. (2017). Reintegration assistance after prison: Follow‐up on the Prison and Probation Service's work with special reintegration assistance measures. The Swedish National Council for Crime Prevention (Brå) –centre for knowledge about crime and crime prevention measures. https://bra.se/bra-in-english/home/publications/archive/publications/2018-02-06-reintegration-assistance-after-prison.html

[cl21255-bib-0010] Burrowes, N. , Disley, E. , Liddle, M. , Maguire, M. , Rubin, J. , Taylor, J. , & Wright, S. (2013). Intermediate outcomes of arts projects: A rapid evidence assessment. National Offender Management Services.

[cl21255-bib-0011] Caulfield, L. , Jolly, A. , Simpson, E. , & Devi‐McGleish, Y. (2022). ‘It's not just music, it helps you from inside': Mixing methods to understand the impact of music on young people in contact with the criminal justice system. Youth Justice, 22 (1), 67–84.

[cl21255-bib-0012] Central Statistics Office . (2016). *Prison Recidivism 2010 cohort*. https://www.cso.ie/en/releasesandpublications/er/prir/prisonrecidivism2010cohort/

[cl21255-bib-0013] Chang, Z. , Larsson, H. , Lichtenstein, P. , & Fazel, S. (2015). Psychiatric disorders and violent reoffending: A national cohort study of convicted prisoners in Sweden. The Lancet Psychiatry, 2(10), 891–900. 26342957 10.1016/S2215-0366(15)00234-5 PMC4629414

[cl21255-bib-0014] Cheliotis, L. , & Jordanoska, A. (2016). The arts of desistance: Assessing the role of arts‐based programmes in reducing reoffending. Howard Journal of Crime and Justice, 55(1–2), 25–41.

[cl21255-bib-0015] Clean Break . (2017). *Clean Break's education programme, theory of change, and literature review*. Arts Council England. https://www.cleanbreak.org.uk/media/uploads/theory_of_change_final_april_2017.pdf

[cl21255-bib-0016] Clift, S. , Morrison, I. , Skingley, A. , Page, S. , Coulton, S. , Treadwell, P. , Vella‐Burrows, T. , Salisbury, I. , & Shipton, M. (2013). *An evaluation of community singing for people with COPD (Chronic Obstructive Pulmonary Disease)—Final report*. https://www.canterbury.ac.uk/medicine-health-and-social-care/sidney-de-haan-research-centre/documents/research/Clift-Morrison-Skingley-Page-Coulton-Treadwell-VellaBurrows-Salisbury-Shipton-Evaluation-of-Community-Singing-for-People-with-COPD-FINAL.pdf

[cl21255-bib-0017] Coates, S. (2016). *Unlocking potential: A review of education in prisons*. Ministry of Justice. http://assets.publishing.service.gov.uk/government/uploads/system/uploads/attachment_data/file/524013/education-review-report.pdf

[cl21255-bib-0018] Cohen, M. L. (2009). Choral singing and prison inmates: Influences of performing in a prison choir. Journal of Correctional Education, 60(1), 52–65.

[cl21255-bib-0019] Cohen, M. L. (2012). Harmony within the walls: Perceptions of worthiness and competence in a community prison choir. International Journal of Music Education, 30(1), 46–56.

[cl21255-bib-0020] Coholic, D. , Schinke, R. , Oghene, O. , Dano, K. , Jago, M. , McAlister, H. , & Grynspan, P. (2020). Arts‐based interventions for youth with mental health challenges. Journal of Social Work, 20(3), 269–286.

[cl21255-bib-0021] Coutinho, B. naV. , Hansen, A. L. , Waage, L. , Hillecke, T. K. , & Koenig, J. (2015). Music making interventions with adults in the forensic setting—A systematic review of the literature—Part I: Group interventions. Music and Medicine, 7(3), 40–53.

[cl21255-bib-0022] Coutinho, B. V. , Hansen, A. L. , Waage, L. , Hillecke, T. K. , & Koenig, J. (2015). Music making interventions with adults in the forensic setting—A systematic review of the literature—Part II: Case studies and good vibrations. Music and Medicine, 7(4), 50–71.

[cl21255-bib-0023] Crossick, G. , & Kaszynska, P. (2016). Understanding the value of arts and culture: The AHRC Cultural Value Project. Arts and Humanities Research Council.

[cl21255-bib-0024] Cucca, A. , Di Rocco, A. , Acosta, I. , Beheshti, M. , Berberian, M. , Bertisch, H. C. , Droby, A. , Ettinger, T. , Hudson, T. E. , Inglese, M. , Jung, Y. J. , Mania, D. F. , Quartarone, A. , Rizzo, J. R. , Sharma, K. , Feigin, A. , Biagioni, M. C. , & Ghilardi, M. F. (2021). Art therapy for Parkinson's disease. Parkinsonism and Related Disorders, 84, 148–154. 33526323 10.1016/j.parkreldis.2021.01.013

[cl21255-bib-0025] Davey, L. , Day, A. , & Balfour, M. (2015). Performing desistance: how might theories of desistance from crime help us to understand the possibilities of prison theatre? International Journal of Offender Therapy and Comparative Criminology, 58(8), 798–809. 10.1177/0306624X14529728 24709832

[cl21255-bib-0026] De Claire, K. , & Dixon, L. (2017). The effects of prison visits from family members on prisoners' well‐being, prison rule breaking, and recidivism: A review of research since 1991. Trauma, Violence, and Abuse, 18(2), 185–199. 10.1177/1524838015603209 26330175

[cl21255-bib-0027] Digital Solution Foundry of EPPI Centre . (2020). *EPPI Mapper Version 1.2.5* [Computer program]. Digital Solution Foundary and EPPI Centre. EPPI Centre, UCL Social Research Institute, University College London.

[cl21255-bib-0028] Duque, M. , & Mcknight, A. (2019). *Understanding the relationship between inequalities and poverty: Mechanisms associated with crime, the legal system and punitive sanctions*. http://sticerd.lse.ac.uk/case

[cl21255-bib-0029] Durose, M. R. , Cooper, A. D. , & Snyder, H. N. (2014). Bureau of Justice Statistics (BJS)—Recidivism of Prisoners Released in 30 States in 2005: Patterns from 2005 to 2010—Update . https://www.bjs.gov/index.cfm?ty=pbdetail&iid=4986

[cl21255-bib-0030] Eaglesham, P. , Gallagher, L. , Kennedy, W. , Macmaster, B. , Tobin, J. , Conaglen, P. , Cosgrove, K. , Dryden, R. , Everington, T. , Hetherington, K. , Fraser, A. , Graham, L. , Hogg, E. , Hardie, S. , Jackson, T. , Loucks, N. , McPherson, M. , McGowan, M. , Merrin, N. , & Young, F. (2017). *Reducing offending, reducing inequalities. Achieving 'better health, better lives' through community justice*. http://www.healthscotland.scot/media/1528/reducing-offending-reducing-inequalities_aug_english.pdf

[cl21255-bib-0031] Fazel, S. , Grann, M. , Kling, B. , & Hawton, K. (2011). Prison suicide in 12 countries: An ecological study of 861 suicides during 2003‐2007. Social Psychiatry and Psychiatric Epidemiology, 46(3), 191–195. 20140663 10.1007/s00127-010-0184-4

[cl21255-bib-0032] Fazel, S. , Taanvi, R. , & Keith, H. (2017). Suicide in prisons: An international study of prevalence and contributory factors. The Lancet Psychiatry, 4(12), 946–952. 29179937 10.1016/S2215-0366(17)30430-3 PMC6066090

[cl21255-bib-0033] George, O. , & Kasinathan, J. (2015). Mural art therapy for young offenders hospitalised with a mental illness. Australasian Psychiatry, 23(1), 49–53. 25519999 10.1177/1039856214563852

[cl21255-bib-0034] Global Prison Trends . (2020). *Global prison trends—Special focus pull‐out section: Alternatives to imprisonment*. Penal Reform International and Thailand Institute of Justice. https://cdn.penalreform.org/wp-content/uploads/2020/05/Global-Prison-Trends-2020-Penal-Reform-International-Second-Edition.pdf

[cl21255-bib-0035] Hewson, T. , Shepherd, A. , Hard, J. , & Shaw, J. (2020). Effects of the COVID‐19 pandemic on the mental health of prisoners. The Lancet Psychiatry, 7(7), 568–570. 32563298 10.1016/S2215-0366(20)30241-8 PMC7302764

[cl21255-bib-0036] Higgins, J. P. T. , Savović, J. , Page, M. J. , Elbers, R. G. , & Sterne, J. A. C. (2019). *Chapter 8: Assessing risk of bias in a randomized trial | Cochrane Training*. The Cochrane Collaboration. https://training.cochrane.org/handbook/current/chapter-08

[cl21255-bib-0037] Houchin, R. (2005). *Social exclusion and imprisonment in Scotland: A report*. https://www.scotpho.org.uk/media/1847/social-exclusion-and-imprisonment-in-scotland-.pdf

[cl21255-bib-0038] House of Commons Health and Social Care Committee Prison Health . (2018). *Twelfth Report of Session 2017–19*. Prison Health 2018 (HC 963), pp. 1–73.

[cl21255-bib-0039] Hu, J. , Lu, L. , Zhu, M. , Li, Z. , Wang, C. , Gu, S. , Meng, L. , Liu, H. , Hu, Z. , & Xu, Y. (2020). The cognitive and socioemotional effects of a short‐term light painting intervention. Social Behavior and Personality, 48(8), e9270.

[cl21255-bib-0040] Jabbari, M. , & Dadvar, A. (2018). The role of theater of the oppressed on correction and rehabilitation of prisoners (case study: Yazd Province Prisons). Journal of History Culture and Art Research, 7(3), 521.

[cl21255-bib-0041] Keenan, C. , & White, H. (2018). *Qualitative Critical Appraisal Tool* (unpublished).

[cl21255-bib-0042] Khadar, M. G. , Babapour, J. , & Sabourimoghaddam, H. (2013). The effect of art therapy based on painting therapy in reducing symptoms of separation anxiety disorder (SAD) in elementary school boys. Procedia—Social and Behavioral Sciences, 84, 1697–1703.

[cl21255-bib-0043] Kimport, E. R. , & Hartzell, E. (2015). Clay and anxiety reduction: A one‐group, pretest/posttest design with patients on a psychiatric unit. Art Therapy, 32(4), 184–189.

[cl21255-bib-0044] Lawrence, O. G. , Vanchieri, C. , & Pope, A. (2007). Ethical considerations for research involving prisoners. National Academies Press. 20669441

[cl21255-bib-0045] Liddle, M. , Disley, E. , Maguire, M. , Meek, R. , & Renshaw, J. (2019). Intermediate outcomes measurement instrument (IOMI) toolkit: Guidance notes (pp. 1–67). Ministry of Justice Analytical Series.

[cl21255-bib-0046] Liu, H. , Song, M. , Zhai, Z. H. , Shi, R. , Jie , & Zhou, X. L. (2019). Group singing improves depression and life quality in patients with stable COPD: A randomized community‐based trial in China. Quality of Life Research, 28(3), 725–735. 30612266 10.1007/s11136-018-2063-5 PMC6394522

[cl21255-bib-0047] Maguire, M. , Disley, E. , Liddle, M. , Meek, R. , & Burrowes, N. (2019). *Developing a toolkit to measure intermediate outcomes to reduce reoffending from arts and mentoring interventions*. Ministry of Justice. https://assets.publishing.service.gov.uk/government/uploads/system/uploads/attachment_data/file/787767/intermediate-outcomes-toolkit-report.pdf

[cl21255-bib-0048] Maruna, S. , & Farrall, S. (2004). Desistance from crime: A theorectical reformulation. In *Soziologie der Kriminalität* (Vol. 43, pp. 171–194). Kölner Zeitschrift für Soziologie und Sozialpsychologie.

[cl21255-bib-0049] Maruna, S. (2001). *Making good: How ex‐convicts reform and rebuild their lives*. American Psychological Association.

[cl21255-bib-0050] McAvinchey, C. (2017). The performance of prison theatre practices: Questioning the evidence. In M. Reason , & N. Rowe (Eds.), Applied practice: Evidence and impact in theatre, music and art (pp. 139–155). Bloomsbury.

[cl21255-bib-0051] McDonald, A. , & Holttum, S. (2020). Primary‐school‐based art therapy: A mixed methods comparison study on children's classroom learning. International Journal of Art Therapy, 25(3), 119–131.

[cl21255-bib-0052] Angus McLewin Associates . (2011). *Arts Alliance: Evidence Library*. National Criminal Arts Alliance. http://www.artsevidence.org.uk/media/uploads/evaluation-downloads/arts-alliance-evidence-library-.pdf

[cl21255-bib-0053] McNeill, F. , Anderson, K. , Colvin, S. , Overy, K. , Sparks, R. , & Tett, L. (2011). Inspiring desistance? Arts projects and ‘what works?’ [‘Kunstprojecten en What Works; een stimulans voor desistance?’]. Justitiele verkenningen, 37(5), 80–101.

[cl21255-bib-0054] McNeill, F. , Farrall, S. , Lightowler, C. , & Maruna, S. (2012). *How and why people stop offending: Discovering desistance*. Insights.

[cl21255-bib-0055] Meekums, B. , & Daniel, J. (2011). Arts with offenders: A literature synthesis. Arts in Psychotherapy, 38(4), 229–238.

[cl21255-bib-0056] Ministry of Justice . (2015). *Policy paper—2010 to government policy: Reoffending and rehabilitation*. https://www.gov.uk/government/publications/2010-to-2015-government-policy-reoffending-and-rehabilitation/2010-to-2015-government-policy-reoffending-and-rehabilitation

[cl21255-bib-0057] National Statistics . (2021). *Safety in Custody Statistics, England and Wales: Deaths in Prison Custody to March, Assaults and Self‐harm to December 2020*. https://www.gov.uk/government/statistics/safety-in-custody-quarterly-update-to-december-2020/safety-in-custody-statistics-england-and-wales-deaths-in-prison-custody-to-march-2021-assaults-and-self-harm-to-december-2020

[cl21255-bib-0058] National Criminal Justice Arts Alliance . (2021). *Creativity in a restricted regime—Enabling creativity for wellbeing and a continuing rehabilitative culture in criminal justice settings during Covid‐19 and beyond: A guide for prison staff*. https://www.artsincriminaljustice.org.uk/wp-content/uploads//04/Creativity-in-a-restricted-regime-a-guide-for-prison-staff.pdf

[cl21255-bib-0059] Nickeas, S. (2018). *Arts interventions and the desistance process: Agency through art among female offenders during incarceration and upon release*. University of West London.

[cl21255-bib-0060] Parkes, R. , & Bilby, C. (2010). The courage to create: The role of artistic and spiritual activities in prisons. The Howard Journal of Criminal Justice, 49(2), 97–110.

[cl21255-bib-0061] Plant, J. , & Dixon, D. (2019). *Enhancing arts and culture in the criminal justice system—A partnership approach*. Clinks. https://www.artsincriminaljustice.org.uk/wp-content/uploads//06/Enhancing-arts-and-culture-in-the-criminal-justice-system.pdf

[cl21255-bib-0062] Shea, B. J. , Reeves, B. C. , Wells, G. , Thuku, M. , Hamel, C. , Moran, J. , Moher, D. , Tugwell, P. , Welch, V. , Kristjansson, E. , & Henry, D. A. (2017). AMSTAR 2: A critical appraisal tool for systematic reviews that include randomised or non‐randomised studies of healthcare interventions, or both. BMJ, 358, j4008. 28935701 10.1136/bmj.j4008 PMC5833365

[cl21255-bib-0063] Shepherd, S. M. , & Purcell, R. (2015). What are the factors associated with criminal behaviour for young people with mental health problems? Psychiatry, Psychology and Law, 22(6), 869–879.

[cl21255-bib-0064] Shoesmith, E. , Charura, D. , & Surr, C. (2021). Acceptability and feasibility study of a six‐week person‐centred, therapeutic visual art intervention for people with dementia. Arts and Health, 13(3), 296–314. 32744920 10.1080/17533015.2020.1802607

[cl21255-bib-0065] Social Exclusion Unit . (2002). *Reducing re‐offending by ex‐prisoners*. pp. 1–221. http://webarchive.nationalarchives.gov.uk; http:/www.cabinetoffice.gov.uk/media/cabinetoffice/social_exclusion_task_force/assets/publications_1997_to_2006/reducing_summary.pdf

[cl21255-bib-0066] Sterne, J. A. C. , Hernán, M. A. , Reeves, B. C. , Savović, J. , Berkman, N. D. , Viswanathan, M. , Henry, D. , Altman, D. G. , Ansari, M. T. , Boutron, I. , Carpenter, J. R. , Chan, A.‐W. , Churchill, R. , Jonathan, J. D. , Hróbjartsson, A. , Kirkham, J. , Jüni, P. , Loke, Y. K. , Pigott, T. D. , … Higgins, J. P. T. (2016). ROBINS‐I: A tool for assessing risk of bias in non‐randomised studies of interventions. BMJ, 355, 7. 10.1136/bmj.i4919 PMC5062054 27733354

[cl21255-bib-0067] The Forgiveness Project . (2010). *The Forgiveness Project's RESTORE approach, to achieve desistance from crime*. https://www.theforgivenessproject.com/wp-content/uploads/2020/04/Theory-of-Change-Narrative-1.pdf

[cl21255-bib-0068] Thomas, J. , Brunton, J. , & Graziosi, S. (2010). *EPPI Reviewer 4: Software for research synthesis* [Computer program]. Social Science Research Unit, EPPI Centre Software, UCL Institute of Education.

[cl21255-bib-0069] United Nations Office on Drugs and Crime . (2012). *Introductory Handbook on the Prevention of Recidivism and the Social Reintegration of Offenders*. Criminal Justice Handbook Series.

[cl21255-bib-0070] Webster, C. , & Kingston, S. (2014, May). *Poverty and Crime Reviewcrime review* (pp. 1–47). Joseph Rowntree Foundation.

[cl21255-bib-0071] World Health Organisation . (2021). *Prisons and health—Mental health*. https://www.euro.who.int/en/health-topics/health-determinants/prisons-and-health/focus-areas/mental-health

