# Supplementary material for: PROTOCOL: Arts‐based interventions for offenders in secure criminal justice settings to improve rehabilitation outcomes: An evidence and gap map
Source: Campbell Syst Rev. 2022 Aug 23;18(3):e1255. doi: 10.1002/cl2.1255 (PMC9399450; doi:10.1002/cl2.1255)
Supplement: Supplementary file 1 — Supporting information. [file CL2-18-e1255-s001.docx]

Appendices

1 Link to online interactive EGM

Add link at the full report stage

2 Sample Search Strategy - PsycInfo

| 1 | (efficac* or effective* or evaluat* or experiment* or interven* or program* or quasi  or random* or RCT or treat* or trial or therap*).ab,id,sh,ot,ti. |
| --- | --- |
| 2 | exp Art Therapy/ |
| 3 | (art or arts or artistic or dance or dancer* or danced or drama* or film* or music*  or paint* or photograph* or poet* or sculpt* or sing or sings or singer* or sketch* or  song or songs or story or stories or theatr*).ab,id,sh,ot,ti. |
| 4 | 2 or 3 |
| 5 | exp Prisoners/ or exp Criminal Offenders/ |
| 6 | exp Correctional Institutions/ or exp Prison/ or exp Criminal Rehabilitation/ or exp  Incarceration/ |
| 7 | (corrections or correctional or criminal* or custody or custodial or detention* or  detain* or felon* or forensic or gaol* or incarcerat* or inmate* or imprison* or jail* or  offender* or prison*).ab,id,sh,ot,ti. |
| 8 | exp Psychiatric Patients/ or exp Forensic Psychology/ or exp Forensic  Psychiatry/ |
| 9 | ((secur* or forensic or psychiatr* or locked) adj3 (facility or facilities or unit or  units or hospital* or ward or center* or centre* or institution*)).ab,id,ot,sh,ti. |
| 10 | 5 or 6 or 7 or 8 or 9 |
| 11 | 1 and 4 and 10 |

## 3 Coding Categories

[Enter text here]

| **Study Design** | | |
| --- | --- | --- |
| **Area** | **Code** | **Comment** |
| ***Descriptive Information*** | Title  Author citation  Publication date  URL  Volume No.  Issue No.  Publication type (i.e. peer-review, technical report, unpublished, thesis/dissertation, other) | Open |
| ***Study Funding*** | Internal  External  Charitable  National  Other:  Not mentioned/No funding |  |
| ***Study design*** | Systematic Review  RCT  Non-RCT  Mixed methods  Qualitative |  |
| ***Status of study*** | Completed  Ongoing  Unclear |  |
| ***Systematicpractitioner review quality***  ***(AMSTAR)*** | Low (AMSTAR 0-3)  Moderate (AMSTAR 4-7)  High (AMSTAR 8-11) |  |
| ***Primary study quality***  ***(Cochrane Risk of Bias 2)*** | Low  Moderate  High |  |
| ***Qualitative Critical Appraisal Tool*** | Low  Medium  High |  |
| **Population** | | |
| **Area** | **Code** | **Comment** |
| ***Equity Characteristics Noted*** | Yes  No |  |
| ***Age*** | Juvenile (under 18)  Adult (19-65)  Old age (65+) |  |
| ***Gender*** | Male  Female  Mixed  Undefined |  |
| ***Types of populations*** | Pregnant women  People with a learning disability  People with a mental health need  Ethnic minorities  Rural populations  LGBTQ+  Other:  Mixed |  |
| ***Mental health diagnosis*** | Psychotic  Neurotic, stress-related & somatoform disorder)  Mood (affective) disorder  Personality disorder  Organic mental disorder  Other:  Mixed  Unclear/not applicable |  |
| ***Comorbidity*** | Substance abuse  Mental health  Other:  Unclear/not applicable |  |
| **Intervention** | | |
| **Area** | **Code** | **Comment** |
| ***Type of art intervention*** | Music  Creative writing  Theatre performance  Visual arts  Movement  Multi-arts  Unclear |  |
| ***Length of intervention*** | Less than one month  1-3 months  3-6 months  Longer than six months  Unclear |  |
| ***Delivery*** | Individual  Group  Online/Distance  Other: _____________  Mixed  Unclear |  |
| ***Length of Follow-up*** | Less than one month  1-3 months  3-6 months  6-9 months  9-12 months  Longer than one year  Unclear |  |
| ***Art Facilitator*** | Arts practicioner  Correctional staff  Medical staff  Peer-led  Other:  Mixed  Unclear |  |
| ***Attendance Level*** | Low  Moderate  High  Unclear |  |
| **Setting** |  |  |
| **Area** | **Code** | **Comment** |
| ***Geographic Information*** | World Bank Regions:  South Asia  Sub-Saharan Africa  East Asia & Pacific  Europe & Central Asia  Latin America & Caribbean |  |
| ***Institutional Setting*** | Prison  Jail  Youth Offending Centre  Secure Old Age Care Home  Psychiatric hospital  Other:  Mixed  Unclear |  |
| ***Security Level*** | Maximum/High  Medium  Low  Open  Unclear |  |
| ***Intermediate Outcomes*** |  |  |
| **Area** | **Code** | **Comment** |
| ***Personal*** | Resilience  Wellbeing  Agency/self-efficacy  Impulsivity/problem-solving  Motivation to change  Hope  Interpersonal trust  Creative expression |  |
| ***Interpersonal*** | Healthier relationships  Improved teamwork  Flexible thinking  Communication  Decision-making  Pro-social  Problem-solving skills  Creative exchange |  |
| ***Community*** | Feeling valued  Sense of trust & belonging  Stronger networks of support  Contribution to cultural & community life |  |
| **Desistance Outcomes** |  |  |
| **Area** | **Code** | **Comment** |
| ***Institution outcomes*** | Behaviour  Communication  Managing relationships (staff, other inmates, family, etc.)  Other:  Unclear |  |
| ***Intervention Outcome*** | Rehabilitation  Desistance  Behaviour and attitude  Other:  Unclear |  |
